# Supplementary material for: Hidden Burden of a Measles Outbreak Revealed by Genomic and Transmission Models
Source: medRxiv. 2026 Jul 15:2026.07.09.26357695. Preprint. [Version 1] doi: 10.64898/2026.07.09.26357695 (PMC13409256; doi:10.64898/2026.07.09.26357695)
Supplement: Supplementary file 1 [file NIHPP2026.07.09.26357695v1-supplement-1.pdf]

medRxiv preprint doi: https://doi.org/10.64898/2026.07.09.26357695; this version posted July 15, 2026. The copyright holder for this preprint (which was not certified by peer review) is the author/funder, who has granted medRxiv a license to display the preprint in perpetuity.
It is made available under a CC-BY 4.0 International license .

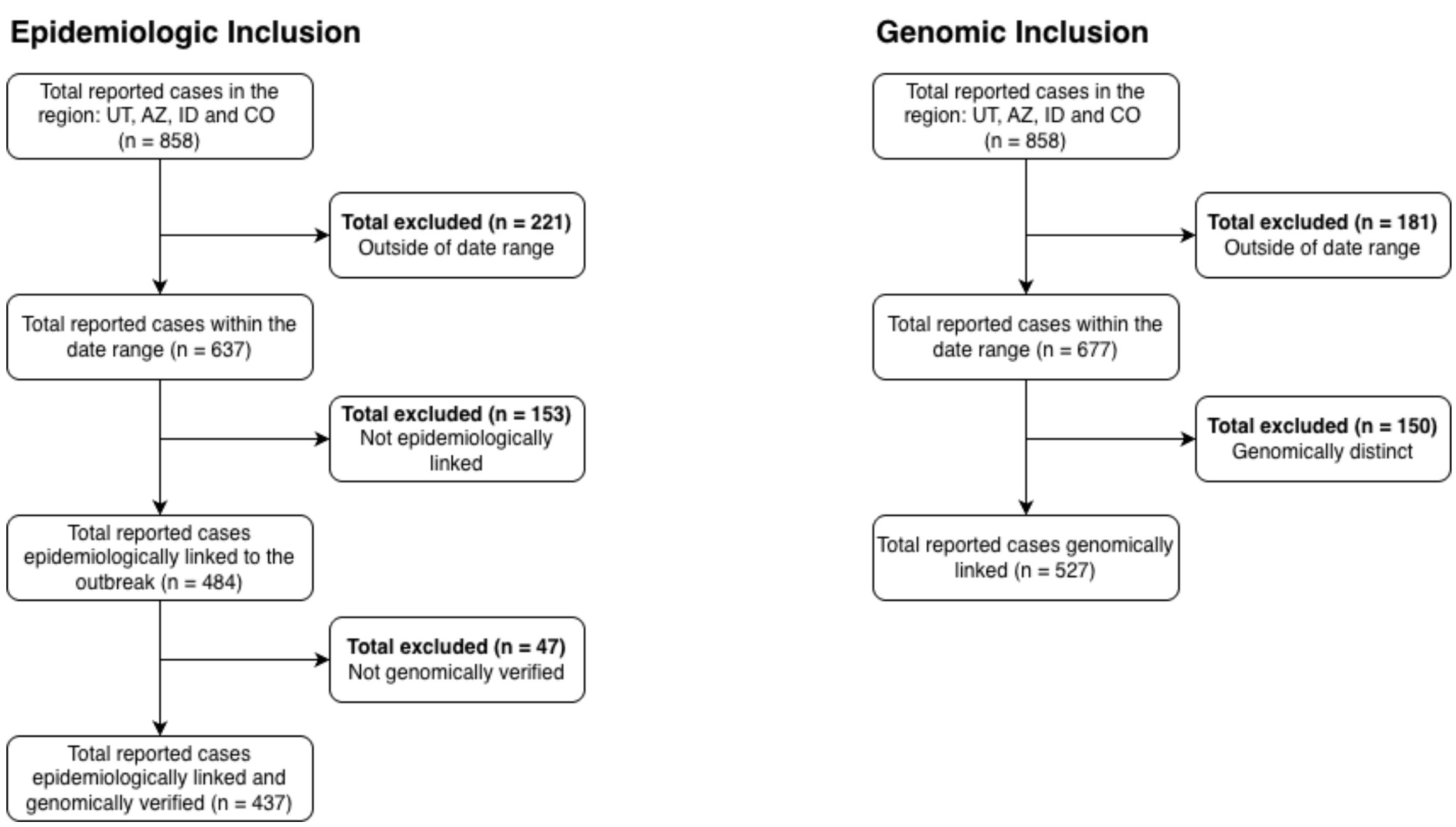

Figure SI1: Diagrams of the inclusion criteria for the phylodynamic analysis for A) epidemiologic inclusion criteria and B) genomic inclusion criteria.

medRxiv preprint doi: https://doi.org/10.64898/2026.07.09.26357695; this version posted July 15, 2026. The copyright holder for this preprint (which was not certified by peer review) is the author/funder, who has granted medRxiv a license to display the preprint in perpetuity.
It is made available under a CC-BY 4.0 International license .

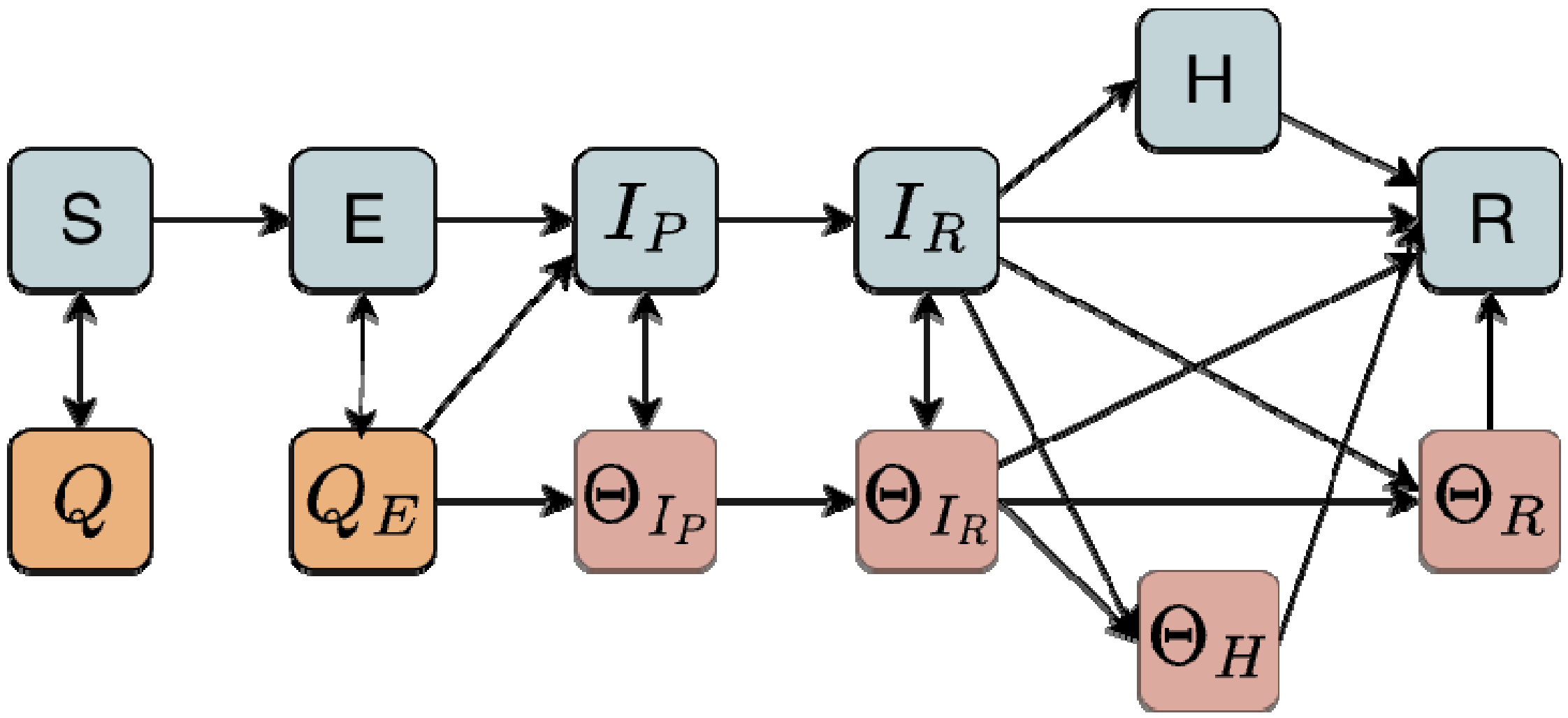

Figure SI3: Model schematic of the measles ABM. The blue shaded boxes indicate the disease progression model with susceptibles (S) becoming exposed (E) progressing to infectious and prodromal (I_P), then becoming infectious with a rash (I_R) finally infections are either hospitalized (H) before they recover (R) or recover directly without hospitalization. The orange shaded boxes indicate quarantine (Q and Q_E) and the red shaded boxes indicate isolated infections ($\square_{IP}$, $\square_{IR}$, $\square_{H}$, and $\square_{R}$). Quarantined susceptibles (Q) can only leave quarantine whereas quarantined exposed infections (Q_E) can either leave quarantine or progress through the disease states while in isolation. Isolated infections similarly can leave isolation or progress through the disease states. Agents move between states at different rates depending on their vaccination status[49]. The model is stochastic except for the number of days agents spend in quarantine (21 days) and isolation (4 days).

medRxiv preprint doi: https://doi.org/10.64898/2026.07.09.26357695; this version posted July 15, 2026. The copyright holder for this preprint (which was not certified by peer review) is the author/funder, who has granted medRxiv a license to display the preprint in perpetuity.
It is made available under a CC-BY 4.0 International license .

Table SI 1: Simulation Parameters for the Agent Based Model

Parameter	Meaning	Value
Contact tracing window	How many days before/after rash onset will be considered for the contact tracing	4.00 days
Contact tracing success rate	The average proportion of cases where a contact will be detected, per event	0.25
Mean days undetected	Average number of days from rash onset to detection	2.00 days
Mean Hospitalization period	Average number of days an infection is hospitalized	7.00 days
Hospitalization rate	Adjusted to match a 10% hospitalization probability	0.037/day
Mean incubation period	Average duration of time from exposure to prodromal symptom onset	12.00 days
Isolation period	Fixed duration of time that infections with rash are isolated	4.00 days
Isolation willingness	Probability of adherence to isolation	0.90
Mean prodromal period	Average duration of non-specific symptoms	4.00 days
Quarantine period	Fixed duration of time that contacts of infected individuals are quarantined	21.00 days
Quarantine willingness	Probability of adherence to quarantine	0.90
Mean rash period	Average duration of rash symptoms	4.00 days
Vaccine efficacy	Effectiveness of the vaccine at mounting an immune response, that is, 97% of vaccinated individuals are totally immune and 3% are totally susceptible	0.97
